# Correction to: Clinical Features, Treatment, and Outcome of Psittacosis Pneumonia: A Multicenter Study

**DOI:** 10.1093/ofid/ofad191

**Published:** 2023-04-12

In the original publication of this article, [Ni Y, Zhong H, Gu Y et al. Clinical Features, Treatment, and Outcome of Psittacosis Pneumonia: A Multicenter Study, Open Forum Infectious Diseases, Volume 10, Issue 2, February 2023, ofac518, https://doi.org/10.1093/ofid/ofac518], affiliations 1 and 10 were partially incorrect and have been revised in the version currently available online. The authors regret this error.

